# LncRNA SAMMSON negatively regulates miR-9-3p in hepatocellular carcinoma cells and has prognostic values

**DOI:** 10.1042/BSR20190615

**Published:** 2019-07-05

**Authors:** Shouzhang Yang, Huajie Cai, Bingren Hu, Jinfu Tu

**Affiliations:** Department of Hepatobiliary Surgery, The First Affiliated Hospital of Wenzhou Medical University, Wenzhou City 325000, Zhejiang Province, P.R. China

**Keywords:** hepatocellular carcinoma, lncRNA SAMMSON, miR-9-3p, survival

## Abstract

In the present study, we investigated the role of lncRNA SAMMSON in hepatocellular carcinoma (HCC). We found that SAMMSON was up-regulated in HCC tissues, and patients with high levels of SAMMSON in HCC tissues had significantly lower overall rate within 5 years after admission. miR-9-3p was down-regulated in HCC tissues and inversely correlated with SAMMSON. SAMMSON expression was not significantly affected by HBV and HCV infections in HCC patients. In HCC cells, SAMMSON overexpression resulted in down-regulated miR-9-3p expression, while miR-9-3p overexpression caused no significant changes in expression levels of SAMMSON. SAMMSON overexpression led to increased, while miR-9-3p overexpression resulted in decreased migration and invasion rates of HCC cells. Therefore, SAMMSON negatively regulated miR-9-3p in HCC cells to promote cancer cell migration and invasion.

## Introduction

Hepatocellular carcinoma (HCC) as the major subtype of liver cancer is characterized by the aggressive nature, high prevalence, high mortality rate, and resistance to currently available chemical drugs [1]. During the development of HCC, portal vein tumor thrombosis frequently occur, leading to intra-hepatic metastasis of HCC cells [2]. In addition, HCC is also prone to long distance metastasis [3,4]. At present, treatment of HCC with radical resection is only limited to the very early clinical stages, while most patients are initially diagnosed at advanced stages [5]. Therefore, the prevention of tumor metastasis and accurate prognosis for patients with metastatic HCC is still critical for the survival of HCC patients.

Studies on the genetics of HCC have identified many genetic alterations involved in HCC, such as the mutations of cancer-related genes and the dysregulated expression of oncogenes or tumor suppressors [6,7]. More and more studies have showed that the altered productions of tumor suppressive or oncogenic protein products are usually caused by the aberrant expression of expression of long non-coding RNAs (lncRNAs, >200 nt), which can regulate gene expression at multiple levels [8,9]. Besides that, lncRNAs can also interact with miRNAs to play their biological roles [10]. LncRNA SAMMSON has recently been characterized as an oncogenic lncRNA in melanoma [11] We performed preliminary microarray analysis and found that SAMMSON was up-regulated in HCC and inversely correlated with miR-9-3p, which plays tumor suppressive role in HCC [12]. We, therefore, carried out this study to explore the possible interaction between SAMMSON and miR-9-3p in HCC.

## Materials and methods

### Research subjects

We selected 70 HCC patients (36 males and 34 females, 32–69 years, 49.1 ± 5.4 years) from the 244 HCC patients who were enrolled by The First Affiliated Hospital of Wenzhou Medical University between April 2010 and December 2014. Inclusion criteria: (1) new HCC cases diagnosed through histopathological tests; (2) patients willing to and completed a 5-year follow-up (telephone or outpatient visit in rare cases). Exclusion criteria: (1) any therapies for any diseases were initiated within 3 months before admission; (2) any other clinical disorders besides HCC were observed; (3) patients died of accidences (such as traffic accidents) and other clinical disorders during follow-up. The existence of HBV and HCV was detected by sensitive PCR. There were 32 cases of HBV positive only, 16 cases of HCV positive only, 10 cases of both HBV and HCV positive, and 12 cases were negative for both HBV and HCV. The 70 patients were classified into AJCC stage I (*n*=14), II (*n*=24), III (*n*=20) and IV (*n*=12). Ethics Committee of the hospital aforementined approved this study. Informed consent was abtained from all the 70 patients.

### Tissue collection

Before the initiation of any therapies, liver biopsy was performed on all HCC patients and adjacent (within 2 cm around tumors) non-cancer and HCC (cancer) tissues were obtained from each patient. All tissues were confirmed by histopathological examinations which were carried out by three experienced pathologists.

### Cells and cell transfections

SNU-182 and SNU-398 human HCC cell lines were included in this study. Cells of these two cell lines were bought from ATCC (U.S.A.). RPMI-1640 Medium (10% FBS) was used as cell culture medium and cell culture conditions were 37°C and 5% CO_2_. SAMMSON was constructed using pcDNA3.1 vector (Sangon, Shanghai, China). Negative control (NC) miRNA and miR-9-3p mimic were from Sigma-Aldrich (U.S.A.). Lipofectamine 2000 reagent (Invitrogen, U.S.A.) was used to transfect 10 nM SAMMSON expression vector (empty vector was used as NC) or 30 nM miR-9-3p mimic (NC miRNA was used as NC). Control (C) group included cells without transfections. Following experiments were performed using SNU-182 and SNU-398 cell collected at 24 h after transfections.

### RT-qPCR

VWR Life Science RiboZol (VWR, U.S.A) was mixed with SNU-182 and SNU-398 cells or tissues (ground in liquid nitrogen) to extract total RNAs. RNA samples were first subjected to DNase I digestion, followed by reverse transcription performed using AMV Reverse Transcriptase (Canvax Biotech, U.S.A). Script One-Step RT-qPCR Kit (Quantabio, Beverly, MA) was used to prepare qPCR mixture with 18S rRNA as endogenous control to analyze the expression of SAMMSON. High Pure miRNA Isolation Kit (Sigma-Aldrich, U.S.A) was used to extract miRNA, followed by reverse transcription using miScript II RT Kit (QIAGEN, Germany). All qPCR reaction mixtures were prepared using mirVana qRT-PCR miRNA Detection Kit (Thermo Fisher Scientific) with U6 as endogenous control to analyze the expression of miR-9-3p. All qPCR reactions were performed three times, and 2^−ΔΔ*C*^_T_ method was used to process all values.

### Cell migration and invasion rate measurement

SNU-182 and SNU-398 cells were collected at 24 h after transfections. RPMI-1640 Medium (1 ml; 1% FBS) was mixed with 3 × 10^4^ cells to prepare single cell suspensions, which were added into upper Transwell chamber. Before invasion assay, upper chamber membrane was coated with Matrigel (356234, Millipore, U.S.A) to mimic *in vivo* invaison. The lower chamber was filled with RPMI-1640 Medium containing 20% FBS. Cell invasion or migration was allowed for 2 h under conditions of 37°C and 5% CO_2_. Finally, 0.5% crystal violet (Sigma-Aldrich, U.S.A) was used to stain upper chamber membranes and stained cells were observed under an optical microscope.

### Statistical analysis

Mean values were calculated using data from three biological replicates of each experiment. Correlations were analyzed by performing linear regression. Based on Youden’s index and SAMMSON level in HCC tissues, 70 HCC patients were grouped into low (*n*=38) and high (*n*=32) level groups. Survival curves were plotted for both groups based on K-M method, and log-rank test was used to compare survival curves. Paired *t* test was used to analyze differences between two types of tissues. One-way ANOVA and Tukey test were used to analyze differences among different patient groups of among different cell transfection groups. *P*<0.05 was the statistically significant level.

## Results

### SAMMSON was up-regulated in HCC tissues but was not affected by HBV and HCV infections

**Figure 1:**
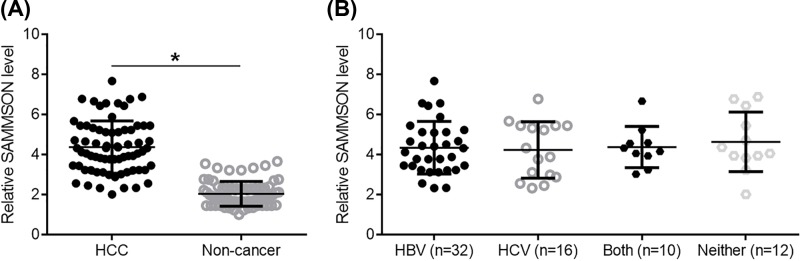
SAMMSON was up-regulated in HCC tissues but was not affected by HBV and HCV infections Expression data compared by performing paired *t* test showed that, comparing with non-cancer tissues, significantly lower expression levels of SAMMSON were observed in HCC tissues. (**A**) (*P<0.05). Comparisons of the expression levels of SAMMSON in HCC tissues were performed by one-way ANOVA and Tukey test. It was observed that expression levels of SAMMSON were not significantly different among these four groups of patients (**B**).

Levels SAMMSON expression in both non-cancer and HCC tissues of HCC patients (*n*=70) were mesaured by performing RT-qPCR. Expression data were compared by performing paired *t* test. Comparing with non-cancer tissues, significantly lower expression levels of SAMMSON were observed in HCC tissues (Figure 1A; *P*<0.05). The existence of HBV and HCV was detected by performing sensitive PCR. Based on the resultes of sensitive PCR, there were 32 cases of HBV positive only (HBV), 16 cases of HCV positive only (HCV), 10 cases of both HBV and HCV positive (Both), and 12 cases were negative for both HBV and HCV (Neither). Comparisons of the expression levels of SAMMSON in HCC tissues were performed by one-way ANOVA and Tukey test. It was observed that expression levels of SAMMSON were not significantly different among these four groups of patients (Figure 1B).

### SAMMSON in HCC tissues were not affected by clinical stages and predicted survival

**Figure 2:**
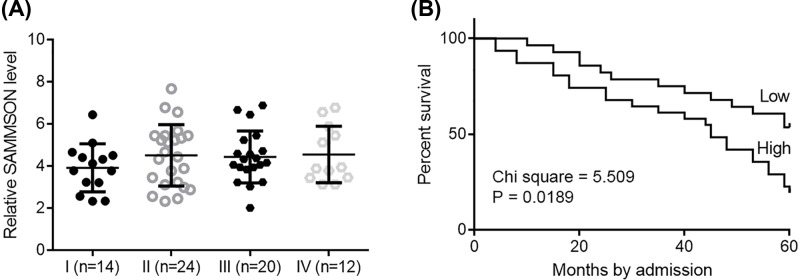
SAMMSON in HCC tissues were not affected by clinical stages and predicted survival One-way ANOVA and Tukey test showed that expression levels of SAMMSON were not significantly different among patients with four different clinical stages. (**A**) Survival curve analysis showed that patients with high levels of SAMMSON in HCC tissues had significantly lower overall rate (**B**).

The 70 patients were classified into AJCC stage I (*n*=14), II (*n*=24), III (*n*=20), and IV (*n*=12). One-way ANOVA and Tukey test showed that expression levels of SAMMSON were not significantly different among patients with different clinical stages (Figure 2A). Based on Youden’s index and SAMMSON level in HCC tissues, 70 HCC patients were grouped into low (*n*=38) and high (*n*=32) level groups. Survival curves were plotted for both groups based on K-M method, and log-rank test was used to compare survival curves. It was observed that patients with high levels of SAMMSON in HCC tissues had significantly lower overall rate within 5 years after admission (Figure 2B).

### SAMMSON was inversely correlated with miR-9-3p

**Figure 3:**
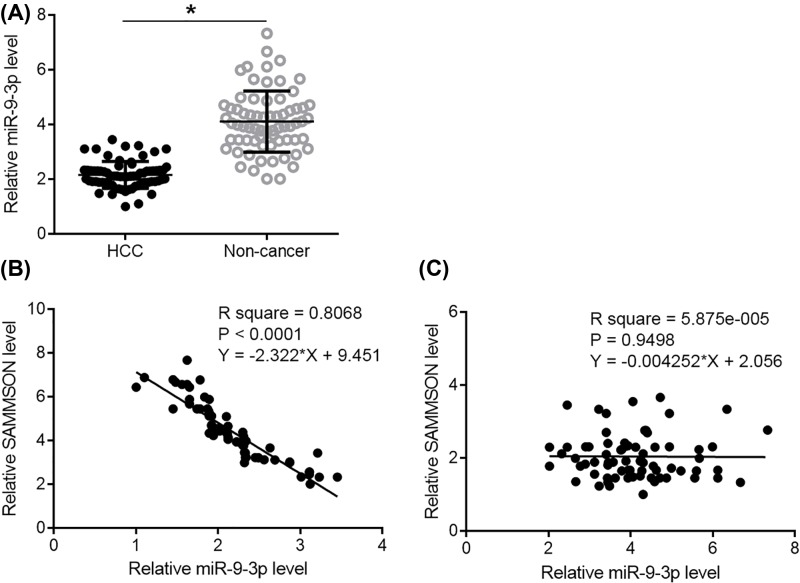
SAMMSON was inversely correlated with miR-9-3p Paired *t* test showed that expression levels of miR-9-3p were significantly lower in HCC tissues comparing withn on-cancer tissues. (**A**) Linear regression showed that SAMMSON and miR-9-3p were inversely and significantly correlated in HCC tissues (**B**) but not in non-cancer tissues (**C**).

RT-qPCR was performed to detect miR-9-3p in two types of tissues. Expression data were analyzed by performing paired *t* test. It was observed that expression levels of miR-9-3p were significantly lower in HCC tissues comparing with non-cancer tissues (Figure 3A, *P*<0.05). The correlation between SAMMSON and miR-9-3p was analyzed by linear regression. In HCC tissues, SAMMSON and miR-9-3p were inversely and significantly correlated (Figure 3B). In non-cancer tissues, SAMMSON and miR-9-3p were not significantly correlated (Figure 3C).

### SAMMSON down-regulated miR-9-3p to suppress HCC cell migration and invasion

**Figure 4:**
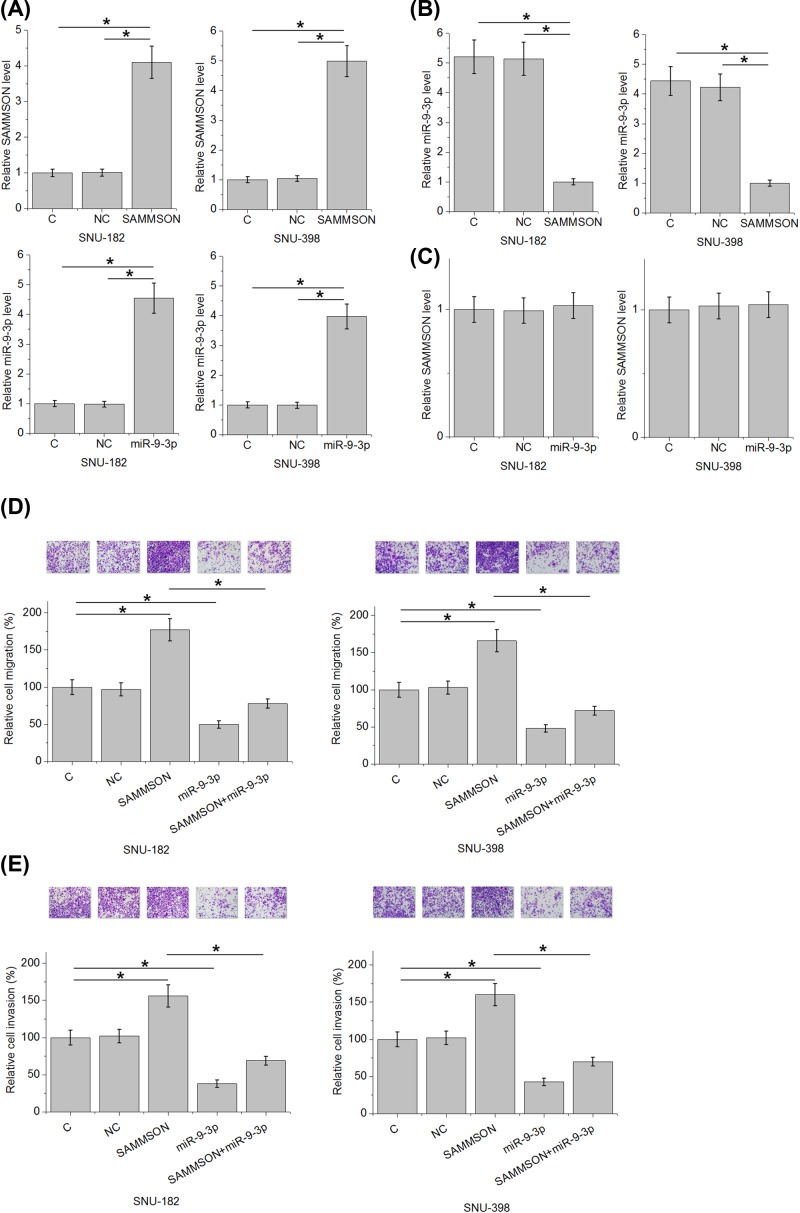
SAMMSON down-regulated miR-9-3p to suppress HCC cell migration and invasion One-way ANOVA and Tukey test showed that expression levels of SAMMSON and miR-9-3p were significantly increased comparing with C and NC at 24 h after transfections (**A**) SAMMSON overexpression resulted in down-regulated miR-9-3p expression (**B**), while miR-9-3p overexpression caused no significantly changes in expression levels of SAMMSON. (**C**) One-way ANOVA and Tukey test showed that SAMMSON overexpression led to increased, while miR-9-3p overexpression resulted in decreased migration (**D**) and invasion (**E**) rates of HCC cells. In addition, miR-9-3p overexpression attenuated the effects of SAMMSON overexpression (*, *P*<0.05).

SAMMSON expression vector and miR-9-3p mimic were transfected into SNU-182 and SNU-398 cells. Comparisons of RT-qPCR data (performed at 24 h after transfection) performed by one-way ANOVA and Tukey test showed that expression levels of SAMMSON and miR-9-3p were significantly increased comparing with C and NC groups (Figure 4A, *P*<0.05). Moreover, SAMMSON overexpression resulted in down-regulated miR-9-3p expression (Figure 4B, *P*<0.05), while miR-9-3p overexpression caused no significantly changes in expression levels of SAMMSON (Figure 4C, *P*<0.05). In addition, analysis of Transwell migration and invasion data by one-way ANOVA and Tukey test showed that SAMMSON overexpression led to increased, while miR-9-3p overexpression resulted in decreased migration (Figure 4D) and invasion (Figure 4E) rates of HCC cells (*P*<0.05). In addition, miR-9-3p overexpression attenuated the effects of SAMMSON overexpression.

## Discussion

SAMMSON is an oncogenic lncRNA in melanoma [11]. The function of this lncRNA in other diseases is unknown. We investigated the role of SAMMSON in melanoma and found that SAMMSON was up-regulated in HCC and had prognostic values. We also showed that SAMMSON has interactions with miR-9-3p in HCC.

Due to the rapid development, tumor metastasis is frequently observed during the development of HCC [5]. Therefore, early diagnosis is still the key for the survival of HCC patients. However, the improvement of HCC diagnosis at initiation stages is limited by the available effective markers [13–15]. In other words, the clinical application of current available early diagnostic biomarkers is limited by their low sensitivity and/or specificity. Therefore, accurate prognosis may be an alternative approach to improve the survival of HCC patients, especially for the ones diagnosed at advanced stages, by guiding the development of individualized treatment for patients with high risk of death in short-term after initial diagnosis. Due to the advantage of non-invasive nature, circulating biomarkers have been widely used in disease prediction [16]. However, our study failed to detect SAMMSON in blood derivatives (serum and plasma) of HCC patients. This is possibly due to the low sensitivity of PCR detection, or because circulating SAMMSON does not exist. However, the present study proved that high SAMMSON expression levels were associated with high mortality rate. In view of the fact that most HCC patients are diagnosed by histopathological examinations, detecting SAMMSON in HCC tissues may be a practical approach to predict the survival of HCC patients.

MiR-9-3p is a well-characterized tumor suppressive miRNA in HCC [12,17]. MiR-9-3p in HCC suppresses the production of many oncogenic proteins, such TAZ and HBGF-5 [12,17]. It has also been reported that miR-9–3p can inhibit epithelial-mesenchymal transition by down-regulating ITGB1, FN1, and ITGAV in nasopharyngeal carcinoma [18]. In the present study, we observed down-regulated miR-9-3p and the promoted cancer cell migration and invasion after miR-9-3p overexpression, further confirming its tumor suppressive role in HCC. We also showed that SAMMSON was likely an upstream inhibitor of miR-9-3p. Therefore, SAMMSON may indirectly promote the production of oncogenic proteins in HCC by down-regulating miR-9-3p. SAMMSON is unlikely a sponge of miR-9-3p due to the lack of promising binding site of miR-9-3p on SAMMSON. Our future study will try to characterize the mechanism that mediates the interaction between miR-9-3p and SAMMSON. In non-cancer tissues, SAMMSON and miR-9-3p were not significantly correlated. Therefore, certain physiological pathways activated during HCC may mediate the interaction between SAMMSON and miR-9-3p.

Our study only detected SAMMSON expression by qPCR. Our future studies will try to perform Northern blot, which is a more sensitive RNA quantitation assay to measure SAMMSON expression levels. LncRNA isoforms may have different roles depending on their subcellular localizations [19]. Our future studies will also investigate the functions of SAMMSON isoforms. At present, our understanding of the function of SAMMSON is still limited. Many oncogenic and tumor suppressive factors, such as Agrin, Rab7, AKT/mTOR pathway, STAT3, and transcription-3 signalling have been identified for HCC [20–24]. Our future studies will study the interaction between SAMMSON and those factors. Taken together, our study first showed that SAMMSON can play an oncogenic role in HCC by down-regulating a miRNA. Our findings enriched our understanding of the pathogenesis of HCC and the functionality of SAMMSON. However, more studies are needed to further elucidate the mechanism that mediates the actions of SAMMSON in HCC. In conclusion, SAMMSON was up-regulated in HCC and promoted HCC cell migration and invasion by down-regulating miR-9-3p.

## Informed Consent

The study followed the tenets of the Declaration of Helsinki, and informed written consent was obtained from all patients and controls after we explained the nature and possible consequences of the study.

## Abbreviations

AJCC: American Joint Committee on Cancer
C: control
FBS: fetal bovine serum
HBGF-5: fibroblast growth factor 5
HBV: hepatitis B virus
HCC: hepatocellular carcinoma
HCV: hepatitis C virus
NC: negative control
RT-qPCR: quantitative revese transcription PCR
